# *In-situ* Spectroscopic Techniques as Critical Evaluation Tools for Electrochemical Carbon dioxide Reduction: A Mini Review

**DOI:** 10.3389/fchem.2020.00137

**Published:** 2020-03-20

**Authors:** K. S. Adarsh, Naveen Chandrasekaran, Vidhya Chakrapani

**Affiliations:** ^1^CSIR-Central Electrochemical Research Institute, Karaikudi, India; ^2^Howard P. Isermann Department of Chemical and Biological Engineering, Rensselaer Polytechnic Institute, Troy, NY, United States

**Keywords:** electrocatalysts, spectroscopy, CO_2_ reduction, FT-IR, XAS, XPS

## Abstract

Electrocatalysis plays a crucial role in modern electrochemical energy conversion technologies as a greener replacement for conventional fossil fuel-based systems. Catalysts employed for electrochemical conversion reactions are expected to be cheaper, durable, and have a balance of active centers (for absorption of the reactants, intermediates formed during the reactions), porous, and electrically conducting material to facilitate the flow of electrons for real-time applications. Spectroscopic and microscopic studies on the electrode-electrolyte interface may lead to better understanding of the structural and compositional deviations occurring during the course of electrochemical reaction. Researchers have put significant efforts in the past decade toward understanding the mechanistic details of electrochemical reactions which resulted in hyphenation of electrochemical-spectroscopic/microscopic techniques. The hyphenation of diverse electrochemical and conventional microscopic, spectroscopic, and chromatographic techniques, in addition to the elementary pre-screening of electrocatalysts using computational methods, have gained deeper understanding of the electrode-electrolyte interface in terms of activity, selectivity, and durability throughout the reaction process. The focus of this mini review is to summarize the hyphenated electrochemical and non-electrochemical techniques as critical evaluation tools for electrocatalysts in the CO_2_ reduction reaction.

## Introduction

Driving chemical reactions using “*green*” electrons is expected to likely play a key role in attaining sustainable energy conversion and storage technologies in the future (Blanco and Modestino, 2019). Catalysts utilized for electrochemical conversion reactions are anticipated to be inexpensive, stable in harsh acidic and alkaline conditions, poised with active centers (for absorption of the reactants, intermediates formed during the reactions), porous, and electrically conducting material to facilitate the flow of electrons (Rasul et al., 2019; Xu et al., 2019). Also, a good electrocatalyst should have low overpotential, high current density with high selectivity, and excellent durability during the course of the electrochemical reaction. The structure, composition of the electrocatalysts, electrolyte, and binders play pivotal roles in determining the above parameters necessary for successful fabrication of an electrochemical device for real-time largescale industrial applications (Kotrel and Bräuninger, 2008; Zeng and Li, 2015; Franzen et al., 2019). Generally, the most commonly investigated energy conversion reactions such as oxygen evolution (OER) (Sapountzi et al., 2017; Zhang et al., 2018), oxygen reduction (ORR) (Dai et al., 2015; Shao et al., 2016), hydrogen oxidations (Skúlason et al., 2010; Durst et al., 2014); hydrogen evolution reactions (HER) (Eftekhari, 2017) are found to be efficient on precious metals or oxides. On the contrary, electrochemical reduction of CO_2_ (CO_2_RR) has been found to occur efficiently on copper-based catalysts (Sen et al., 2014), but the reaction is highly complex involving multiple electrons and proton transfer to form multiple products (Raciti and Wang, 2018). In comparison to the OER, HER, and ORR processes, the CO_2_RR is more energy consuming. CO_2_ is a linear molecule in which two oxygen atoms are covalently doubly bonded to a single carbon atom. The bonding energy of C=O (E_C = O_) of 187 (2 × 93.5) kcal/mol is much greater than the bonding energies of O=O (E_O = O_) of 116 (2 × 58) kcal/mol and C-C (E_C−C_) of 145 (2 × 72.5) kcal/mol. As a result, CO_2_ is chemically inert; hence its activation requires high energy (Qiao et al., 2016). In general, CO_2_RR is found to occur with adsorption of CO_2_ on a catalytic surface followed by the formation of CO_2_ anion radical ($C{O_{2}}^{-•}$). The intermediates and products (both gas and liquid) are found to vary based on the electrocatalytic surface, electrolyte (aqueous and non-aqueous), and the applied potential. For example, employing aqueous electrolyte leads to the formation of diverse products ranging from alcohols, aldehydes and formic acid, whereas CO_2_RR in non-aqueous electrolyte leads to the formation of CO and oxalate (Oh et al., 2014; Qiao et al., 2016; König et al., 2019; Yu et al., 2019). Technologies for the reduction of carbon foot print are imperative to counter the global warming caused due to CO_2_ emissions from burning of fossil fuels and industrial production of diverse chemicals. The world is expected to emanate 40 Gt/year of CO_2_ into the atmosphere. Thus, it will be vital even if a small fraction of globally produced CO_2_ is captured and utilized for the society in various ways. The thermodynamically stable molecule should be considered as a cheaper source of raw materials for sustainable production of fuels, chemicals, polymers etc. (Alper and Yuksel Orhan, 2017). Even though there are a plethora of reports on the utilization of CO_2_ to value added fuels and chemicals, converting CO_2_ in bulk for chemical synthesis is still very exigent, with very few commercial ventures world over. For example, Liquid Light has setup a plant for a two-step conversion of CO_2_ to ethylene glycol. Mantra has designed reactors for single step conversion of CO_2_ to formic acid. Lanzatech uses biological means to convert CO_2_ to alcohols. Mitsui Chemicals in Japan has anticipated the launch of a commercial plant with a capacity of 600,000 t/years for CO_2_ to methanol conversion. Linde has also launched a dry reforming plant for syn-gas production. Evonik have developed a process for CO_2_ conversion to carbonates. The possibility of converting this abundant waste to useful products has created an avenue of interest from the perspectives of both sustainable energy and environmental decontamination. Chemical fixation of CO_2_ to value products might symbolize an attractive way of reducing emissions of CO_2_ to the atmosphere. Using CO_2_ as a feedstock could be considered a potential replacement for fossil fuels and their derivatives, such as natural gas, carbon monoxide, syn-gas, methanol, and long chain hydrocarbons. Even though the market for some chemicals such as formic acid is relatively small, the market could be augmented by utilizing these products as precursors/building blocks for manufacturing other bulk chemicals such as ethanol. It is important to consider that the source of H_2_ to react with CO_2_ to attain various products should be carefully chosen. To engineer affordable electrochemical energy devices, researchers are investigating the possibilities of employing alternate catalysts based on first row transition metals/oxides and metal-free heteroatom-doped carbon allotropes for the aforementioned reactions (Li et al., 2016; Gao et al., 2017a; Cui et al., 2018; Tang et al., 2019). Although the present electroanalytical techniques combined with the *ex-situ* diffraction, microscopic and spectroscopic techniques could give an estimate of the activity, selectivity, and durability of the electrocatalysts, it may not be sufficient to understand the mechanistic details of the electrochemical reaction at the electrified electrode-electrolyte interface (Jiang et al., 2018; Zhu K. et al., 2019). In the quest to replace noble metal catalysts, the most critical issue is to identify and mimic the role of active centers on alternate catalysts proficiently. Recent advances in the theoretical concepts on electrocatalysis through computational methods such as density functional theory (DFT) calculations and molecular modeling has led to efficient screening of the electrocatalyst (Cao et al., 2013; Eslamibidgoli et al., 2016). On the other hand, sophisticated *in-situ* techniques for online monitoring of electrode-electrolyte interface by combining the electro-analytical tools with the specially designed spectroscopic, microscopic and chromatographic techniques has led to the identification of the compositional, structural/morphological deviations and the intermediates or products formed during the course of the electrochemical reactions (Bandarenka et al., 2014; Ye et al., 2016; Surca et al., 2017). These hyphenated techniques can provide valid experimental substantiation to tackle central issues in catalysis: (a) identification of active sites; (b) geometrical configuration of the reactants and the intermediates on the surface of the catalysts; (c) adsorption and desorption energies of reactants and intermediates; (d) reaction pathways; (e) selectivity toward specific product; (f) local pH at electrode-electrolyte interface in the case of aqueous electrolyte; (g) effect of electrolyte; and (h) stability of the electrocatalyst (Dahn and Mao, 1999; Christensen and Hamnett, 2000; Li et al., 2012; Handoko et al., 2018; Narayanan et al., 2019). Keeping in mind the key factors to be addressed for efficient synthesis and identification of electrocatalysts, this mini-review will focus on important in-operando characterization techniques pertinent to CO_2_RR.

## Reaction Mechanism of CO_2_RR

**Figure 1:**
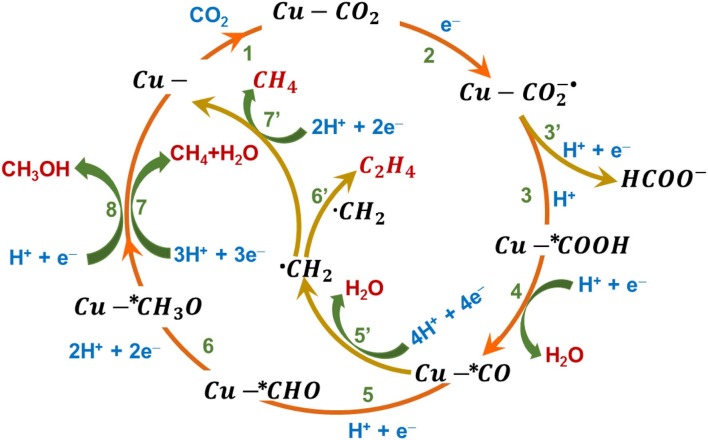
Electrochemical reaction pathways of CO_2_ reduction in aqueous electrolyte system on a Cu model surface.

In aqueous electrolyte systems, CO_2_ reduction yield a variety of C_1_ and C_2_ products. The retention of the CO_2_ anion radical on the electrode surface controls the C-C coupling to obtain higher carbon products. The various reaction pathways are shown schematically in Figure 1. The surface adsorbed CO_2_ molecule accepts an electron when the polarization potential is sufficiently negative to rise the energy of highest occupied molecular orbital (HOMO) of the electrocatalyst above the lowest unoccupied molecular orbital (LUMO) of CO_2_. The single electron reduction of CO_2_ to ${\text{CO}}_{2}^{-\text{.}}$ occurs at a potential of −1.9V vs. the standard hydrogen electrode (SHE) (Reaction-2) (Yu et al., 2019). The CO_2_ anion radical intermediate (${\text{CO}}_{2}^{-\text{.}}$) is highly reactive and, together with a proton from the electrolyte and an electron, produces the most common product formate (HCOO^−^) (Reaction-3′) (Cheng et al., 2016). The competing HER in aqueous electrolytes limits the Faradaic efficiency of CO_2_RR (Ooka et al., 2017). The increase in dissolved concentration of CO_2_ will increase the interaction of CO_2_ with electrode and reduces the extent of HER, hence elevated pressures of CO_2_ is ideal to increase CO_2_RR Faradaic efficiency. The p-block metals such as In, Sn, Hg, Pb, and its oxides have been found to be selective toward formate production (Yu et al., 2019). The other two-electron reduction product of CO_2_, namely CO, can be obtained in an aqueous system through a carboxyl intermediate formed when the proton attacks the ${\text{CO}}_{2}^{-.}$ through the oxygen atom (Reaction-3), the carboxyl intermediate subsequently reacts with another proton and an electron to produce CO and H_2_O (Reaction-4) (Yu et al., 2019). Hori et al. reported a study showing the variation in the obtained products when CO_2_RR occurs on different metal surfaces in different electrolytes (Hori et al., 1994). Their report suggests that the stability of adsorbed ${\text{CO}}_{2}^{-\text{.}}$ plays a crucial role in deciding the product selectivity. The metal surfaces, such as Cu, Au, Zn, Pd, and Ni hold the adsorbed ${\text{CO}}_{2}^{-\text{.}}$ strongly to produce CO preferentially, whereas the metal surfaces that weakly adsorbs ${\text{CO}}_{2}^{-\text{.}}$ leads to the formation of HCOOH/ HCOO^−^. The multiple electron reduction products require thermodynamically less energy, but the process is far more complicated and is limited in terms of kinetics of the reaction. Cu based electrodes are the only reported electrocatalyst with the ability to form C_2_ products (Hori et al., 1997). The mechanisms of these complicated reactions are not very certain; it is proposed that the initially formed CO undergoes further reduction, leading to the formation of different types of intermediates and products. The adsorbed ^*^CO on the electrode surface, reacts with H^+^ and e^−^ to form ^*^CHO intermediate (Reaction-5) which undergoes subsequent reduction by taking up protons and electrons in each step, generating ^*^CH_2_O, ^*^CH_3_O (Reaction-6) (Sun et al., 2007; Qiao et al., 2016). Further reduction of ^*^CH_3_O produces *CH*_3_*OH* when a proton reacts with O of ^*^CH_3_O (Reaction-8), while CH_4_ is produced when a proton attacks the C atom of ^*^CH_3_O instead, and the O atom is combined with two other protons and is removed out as H_2_O (Reaction-7). An alternate pathway is also possible, where the CO reacts with 4H^+^ and 4e^−^ to generate ^*^CH_2_ intermediate (Reaction-5′) and a H_2_O molecule. This ^*^CH_2_ intermediate will get further reduced to CH_4_ (Reaction-7′) or dimerizes to give C_2_ products (Reaction-6′) (Grosjean et al., 2017; Sun et al., 2017; Handoko et al., 2018; Yin et al., 2019; Yu et al., 2019).

CO_2_RR in a non-aqueous electrolyte system is limited with few products but has the added advantages of high CO_2_ solubility and no competing HER, which increases the Faradaic efficiency of the process far above than aqueous systems. Similar to the aqueous system, initially formed reactive species is the ${\text{CO}}_{2}^{-\text{.}}$ anion radical. This anion radical either reacts with dissolved oxygen to produce carbonate or undergoes a disproportionation reaction with a neutral CO_2_ molecule to generate CO and ${\text{CO}}_{3}^{-}$. The increased formation of carbonate beyond a limit of CO_2_ partial pressure can be due to this disproportionation reaction, where there is an enhanced interaction of ${\text{CO}}_{2}^{-\text{.}}$ with neutral CO_2_ molecules (Sullivan et al., 1993; Angamuthu et al., 2010; Qiao et al., 2016; Yu et al., 2019). The dimerization of the ${\text{CO}}_{2}^{-\text{.}}$ in the optimum CO_2_ pressures produces oxalate as the major product.

The bulk properties of the electrode and electrolyte are not always sufficient to describe the reaction and chemical species in the electrode/electrolyte interface. The polarized electrode or the catalyst surface may behave different from the bulk; the unique structure of the interface is the key factor in all electrochemical reactions. The practical difficulty in characterizing the interface during the electrochemical reaction has limited the knowledge on the electrochemical changes taking place on the electrode surface. Hence electrochemists are investigating the possibilities of employing *in-situ* spectro-electrochemical techniques to support the electrochemical measurements. The intermediates formed in the CO_2_RR have been trapped and studied using various *in-situ* spectro-analytical techniques. The online monitoring of live changes happening on the electrode surface and analyzing the electro generated intermediates and products during the electrochemical CO_2_ reduction is inevitable to elucidate mechanistic pathways of this complex reaction.

## *In-situ* Techniques for Electrochemical Co_2_ Reduction

### Infrared Spectroscopy

Infrared (IR) spectroscopy is a powerful analytical tool for monitoring the changes in the chemical bonding of the catalyst. Vibrational modes of chemical bonds that produce difference in the dipole moment during stretching and bending can absorb infrared radiation. IR absorption analysis can provide vital information regarding the structural coordination of the intermediates formed during the reaction (Smith, 2011). Different methods have been employed for the IR absorption measurements on solid samples. The four usual modes of measurements are transmission, diffuse reflectance, attenuated total reflection (ATR) and reflection-absorption, among this ATR mode of measurement is ideal to study electrode surface (Yajima et al., 2004). ATR mode makes use of an ATR crystal made of IR transparent high refractive material. The incident IR radiation with a suitable angle of incidence is reflected at the internal face of the crystal, and the evanescent wave that penetrates the crystal face will interact with the working electrode fabricated as a thin film on the flat face of the cell window (Zhu S. et al., 2019). This wave irradiates the chemical species formed on the vicinity of the electrode surface. ATR working mode with metal electrodes like, Au, Pt has the advantage of surface enhanced IR absorption (SEIRA) which can increase the sensitivity of the method to detect molecules even in trace amounts (Neubrech et al., 2017). Papasizza and Cuesta studied the electrochemical reduction of CO_2_ using ATR-SEIRAS measurements on a gold electrode surface (Papasizza and Cuesta, 2018). Their results proved the formation of electrogenerated (adsorbed) ^*^CO, and also differentiated the CO adsorbed from a CO-saturated electrolyte solution and that produced electrochemically. The study also provided insights into the coordination modes of CO on the Au surface with the observation of separate vibrational bands for linearly coordinated CO (CO_L_) around 1,910 cm^−1^ and a shoulder band observed around 1,750 cm^−1^ corresponding to the bridge coordinated CO (CO_B_)_._ The study reported the formation of only a CO_L_ related band during initial stages and the subsequent formation of a CO_B_ related band occurring only after ~7 min of initiation, which proves that the coordination of electrogenerated CO to the Au surface is through the linear mode. A study from Ito (Oda et al., 1996) showed the electrochemical reduction of CO_2_ and adsorption of CO on a polycrystalline Cu and Ag. They observed the presence of linearly coordinated CO formed during the electrochemical reduction of CO_2_ on both Cu and Ag. They investigated the desorption of CO from the metal surfaces on positive potential sweep and found that the CO desorption was observed from Ag, but not from Cu, which proves the strong adsorption of CO on Cu (not strong enough to be poisoning) than Ag. This result supports the mechanism that higher carbon products formed on Cu is because of the reduction of electrochemically formed adsorbed CO. Wuttig et al. also carried out research on the adsorption of CO on Cu but at varying pH of the electrolyte. They observed an increase in the coverage of adsorbed CO at alkaline pH, notably from pH 6.9–10.1, and a decrease in the coverage at pH higher than 10.1. This finding was related to the observation that the reduction of CO_2_ to higher-order hydrocarbons was generally facile in alkaline media, and that the electrogenerated adsorbed CO further reduces to produce hydrocarbon products (Wuttig et al., 2016).

In addition to the type of metal used as electrocatalysts, studies by Iwasita et al. showed that the activity and selectivity toward CO_2_RR also strongly depend on the orientation of electrocatalytic surface. Studies on Pt surfaces have shown that Pt(110) is the most active, followed by Pt(100) and Pt(111) surfaces (Rodes et al., 1994a,b,c). The differences in the activity were attributed to differences in the type of adsorbed species. In highly active Pt(110) surface, linearly-bonded CO and traces of multibonded CO, with bands at around 2,056 and 1,817 cm^−1^, were observed as the major adsorbates, while the Pt(100) surface showed mainly multibonded CO. No bands in the frequency region 2,100–1,800 cm^−1^ related to the formation of CO-like species was observed on Pt(111).

Advancements in the ATR-SEIRAS technique over the years have led to studies involving the real catalytic systems apart from the model electrode surfaces such as Au, Pt, Ag. Jiang et al. reported a study of CO_2_RR on a Li electrochemically tuned ZnO nanoparticle catalytic system using ATR-SEIRAS. The setup consisted of the catalyst coated on Au under-film on top of a Si ATR crystal (Jiang et al., 2017). Results showed that the catalyst was selective toward CO production with a Faradaic efficiency ~91%, as measured through gas chromatographic analysis. They observed negative intensity peaks ~2,345 and ~1,520–1,544 cm^−1^ which corresponded to the consumption of adsorbed ${\text{CO}}_{2}^{\text{a}}$ and the depletion of carboxyl intermediate ^*^COO^−^, respectively. No peak featuring the adsorption of CO in the region 1,850–2,200 cm^−1^ was identified, which showed that as produced CO immediately desorbed from the Zn surface. The ATR-IR results show that CO is formed through the carboxyl intermediate (^*^COO^−^), as the consumption of ^*^COO^−^ was evident from its negative peak intensity. Another study on the Pd/C catalytic system observed the formation of ^*^CO, ^*^OCHO, and ^*^COOH intermediates (Gao et al., 2017b). Gong et al. studied the role of surface hydroxyls present on the SnO_x_ catalyst toward electroreduction of CO_2_. Using ATR-SEIRAS, they observed the formation of *H*_2_*CO*_3_ on the surface of the catalyst with peaks at 1,498 and 1,435 cm^−1^. Their results showed that an intermediate percentage of surface hydroxyl could promote the adsorption CO_2_ as H_2_CO_3_ through hydrogen bonding, and that *H*_2_*CO*_3_ was found in equilibrium CO_2_. Higher surface coverage of hydroxyls, on the other hand, has a detrimental effect on the efficiency, as it reduces the number of available active sites for CO_2_RR (Deng et al., 2019). Bicarbonate ions in electrolyte solutions are considered to play the role as a proton source and pH buffer. However, through *in-situ* fast ATR-SEIRAS measurements on a Cu surface along with isotopically labeled electrolyte species, Zhu and coworkers showed that bicarbonate ions are the primary CO_2_ source and mediators instead of the free CO_2_ during CO_2_RR (Zhu et al., 2017). They observed the formation of a CO intermediate with a peak at 2,070 cm^−1^ in an Ar-saturated KHCO_3_ solution. Given that Cu-H bonds also have characteristic peaks in the same spectral region, they confirmed that the band indeed corresponded to surface-bonded CO through measurements with isotopically labeled *KT*^13^*CO*_3_. As expected, they observed a red-shift of the peak of ~50 cm^−^ from 2,070 to ~2,022 cm^−1^ consistent with harmonic oscillator model. Comparative measurements were performed in ^12^CO_2_-saturated KH^12^CO_3_ and ^12^CO_2_-saturated KH^13^CO_3_ solutions. In ^12^CO_2_-saturated KH^12^CO_3_, a broad absorbance peak corresponding to ^12^CO at ~2,040 cm^−1^ was observed that blue shifted with time to ~2,060 cm^−1^ along with a negative increase in the absorbance peak at 2,343 cm^−1^, corresponding to the depletion of solution ^12^CO_2_. In KH^13^CO_3_ solution, the formation of the absorbance peak at ~1,992 cm^−1^ that blue shifted with time confirmed the formation of CO. Concurrently, they observed a negative increase in the absorbance peak at 2,277 cm^−1^ corresponding to the depletion of ^13^CO_2_ instead of the ^12^CO_2_ at 2,343 cm^−1^. This observation provided a direct evidence for bicarbonate as the source for the CO_2_RR. The formation of ^13^CO_2_ was attributed to the equilibrium between H^13^${\text{CO}}_{3}^{-}$ and water and its subsequent consumption via electrochemical reduction to form ^13^CO. The authors also noted the formation of a peak corresponding to ^12^CO at ~2,040 cm^−1^ with time that indicated the gradual increase in the H^12^${\text{CO}}_{3}^{-}$ at the interface due to the depleting H^13^${\text{CO}}_{3}^{-}$ and the formation of new bicarbonate ions from free solution ^12^CO. Their experiment clearly proved that CO_2_ is not directly diffusing from the bulk to the electrode surface, instead it is in equilibrium with bicarbonate ions which supplies the CO_2_ through following reaction at the interface:


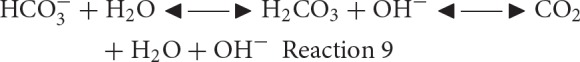


This work provided important insights into the molecular pathway of CO_2_RR on Cu surfaces. Kas et al. summarized that *in-situ* IR techniques are vital probing techniques to expound the reaction mechanisms of chemical and electrochemical CO_2_RR, as the technique can provide extremely valuable information on the nature of reaction intermediates, the significance of double layer effects in manipulating the activity and selectivity, and the nature of the species causing deactivation of the electrocatalysts (Kas et al., 2019).

In addition to the above IR-based techniques, the intermediates of CO_2_RR have also been probed using *in-situ* techniques such as surface-enhanced Raman spectroscopy (SERS) and UV-Vis absorption. An excellent and a more in-depth review of these results can be found in Pérez-Rodríguez et al. (2018).

### X-Ray Based Techniques

#### X-Ray Absorption Spectroscopy

Optical techniques could be well-supported by X-ray techniques in probing the catalyst surface during the course of the electrochemical reaction. In recent years, researchers have exploited X-ray absorption spectroscopy for catalytic studies (Rehr et al., 2010; Singh et al., 2010; Nelson and Miller, 2012). Though X-ray absorption is not very sensitive to lighter elements, it is instrumental to explore the changes happening at the active catalytic sites during the course of catalytic reaction by comparing the X-ray intensities before and after impinging the electrode surface. Key advantages of XAS include the possibilities of measuring accurate particle size of the electrocatalysts and the ability to prod atomic-level structural information on extremely small and complex catalysts (Singh et al., 2010; Dutta et al., 2018; Wang et al., 2019). XAS can provide valuable information on the oxidation state, local coordination environment, and the electronic structure of the material (Bergmann and Roldan Cuenya, 2019). Direct detection of adsorbed species on the catalyst surface is not possible but it can make a difference in the coordination environment of the catalytic sites, and the changes may be reflected in the spectrum (Choi et al., 2017). XAS works based on the principle that, when an incident X-ray radiation having energy greater than the binding energy of core level electrons hits the material, the core level electrons are excited in to the vacuum, producing a strong absorption edge in the spectrum (Handoko et al., 2018; Wang et al., 2019). The changes in the valence electron environment will reflect on the binding energy of core electrons, and thus the chemical changes around an atom can be monitored. All XAS spectra contain four regions: (1) The “pre-edge” region where the incident X-ray energy is less than the binding energy of core electrons. The electrons will not excite to the vacuum level, however some unwanted transitions to higher unfilled or partially filled orbitals take place, which appears as the minor features in the flat region before the strong absorption edge; (2) When the X-ray energy is enough to excite core level electrons in to the unbound states, sudden strong absorption in the spectrum is observed and is called as the X-ray absorption near edge structures (XANES); (3) The region slightly higher in energy than the edge, containing weak absorption humps formed due to the multiple scattering of ejected low energy photoelectrons by the first and higher coordination shells, termed as near edge X-ray absorption fine structure (NEXAFS); and (4) The X-ray energy region starts around 50 eV extended up to 1,000 eV above the edge forms the extended X-ray absorption fine structures (EXAFS). As the energy of ejected electron in this region is much higher, they will undergo scattering (predominantly single scattering) with nearest neighboring atoms. EXAFS region features information on the bond length and the coordination of atoms (Bunker, 2010). EXAFS analysis with an incident angle at 45° can penetrate into the sample >10 μm, and, in order to make it more sensitive toward the surface features, a grazing angle mode can be used (Firet et al., 2019). XAS measurements can usually be done in three different ways using (1) Transmission mode, which measures the difference in the intensity of absorbed and transmitted X-ray radiation. Concentrated homogeneous samples can be measured in this mode. (2) Florescence mode, where intensity of the emitted X-rays from the elements are measured. Because of the self-absorption effect, diluted samples are recommended, particularly for non-homogenous samples; and (3) Photoelectron mode, where, apart from measuring the intensities of X-rays, this third mode measures the energies of ejected photoelectrons. Since the mean free path of the ejected electrons is small, this mode is quite surface sensitive, while the other measurement modes are sensitive to the bulk (Wang et al., 2019).

Wu et al. studied the Zn-porphyrin systems for the electrochemical CO_2_RR to CO through XAS measurements (Wu et al., 2017). Their *in-situ* XAS analysis showed no characteristic changes to the Zn oxidation state, but dissimilarities were observed in the EXAFS region of Zn, which were attributed to the reduction of the porphyrin ligand or the coordination of molecules to the Zn center. The study proposed that porphyrin ligand act as the redox center for the CO_2_RR. Weng et al. reported the selective electrochemical CORR to methane using a copper (II) pthalocyanine catalyst. *In-situ* XAS study proved that the Cu nanocluster like structures formed act as the catalytic reactive sites (Weng et al., 2018). Their XAS measurements revealed the reversible change in the oxidation state of Cu center. The EXAFS analysis displayed the reversible coordination environment around the Cu atom between the forward and reverse scans. Since XAS is not limited to structural examinations, the XAS signs could be used to build the three-dimensional chemical tomography of electrocatalyst during the electrochemical reaction, which may provide insights on the stability of the electrocatalyst.

Genovese et al. performed an *in-operando* XAS study on the variation of catalytic activity of CO_2_RR on Fe-N/C (pyridine-like N functionalized carbon) and Fe-O/C (carboxylic O) functionalized carbon) catalysts. The Fe-N/C catalyst showed excellent CO_2_RR activity with ~61% and ~36.5% Faradaic efficiency toward CH_3_COOH and HCOOH, respectively, whereas the catalyst Fe-O/C was found to be ~95% efficient toward hydrogen evolution. Investigation with operando XAS revealed that Fe^2+^ is stabilized against further reduction to Fe^0^ by the N-dopants. Since this stabilization was not found due to the non-availability of N atoms, Fe-O/C catalyst displayed total reduction to Fe^0^ making it active toward HER rather than CO_2_RR (Genovese et al., 2018). XAS was also employed to investigate the formation of nano-electrocatalytic systems. Roberts et al. investigated the electrochemical generation of copper nanocube catalyst that is active for the electroreduction of CO_2_ to ethylene. The Cu- K edge XAS analysis identified the formation of Cu_2_O intermediate, with the transformation of Cu_2_O to Cu(II)-carbonate/hydroxide species when the potential was increased anodically. Both the Cu(I) and Cu(II) species reduce to metallic Cu during the cathodic polarization. The Cu_2_O and Cu(II)-carbonate/hydroxide derived metallic Cu-nanocubes showed similar activity toward CO_2_RR, suggesting a low significance of the precursor used for the preparation of metallic Cu for its activity toward CO_2_RR (Eilert et al., 2016). Velasco-Vélez et al. hyphenated XAS and *in situ* micro-reactors, which unraveled the variation in the complex electronic structure of the copper oxide catalysts at various stages. It was identified that the surface and bulk properties of the copper oxide catalysts are subjugated by the development of copper carbonates on the cupric oxide surfaces. This led to passivation of the catalyst by impeding the charge transport resulting in formation of CO, and subsequently followed by hydrogenation into C1 and C2 products (Velasco-Vélez et al., 2019). Grosse et al., studied the chemical state and the catalytic selectivity of Cu nanocubes during electrochemical CO_2_RR and using XAFS coupled with wave-let transform and found that no Cu_2_O species remain after 1 h of CO_2_RR either at the surface or in sub-surface regions (Grosse et al., 2018).

An *operando* EXAFS study by Firet et al. on the Ag catalysts demonstrated the crucial role played by weakly bonded atomic oxygen present on the Ag surface for the high CO_2_ reduction reactivity toward CO, which prior XPS studies were unable to reveal due to the facile desorption of weakly bonded oxygen under the ultrahigh vacuum conditions used during XPS measurements (Firet et al., 2019). Roldan et al., studied the interface electrochemistry of SnO_2_NPs@rGO catalyst for CO_2_RR. Sn K edge XANE measurements were performed at different cathodic potentials. Results showed that at −1.15 V (vs. Ag/AgCl), the Sn retains its original (IV) oxidation state. Upon further stepping down at −1.5 V, the white line intensity of Sn K edge absorption was found to decrease, and which continued to decrease with further decrease in potential up to−1.7 V. Decrease in the white line intensity was followed by the shift of K edge absorption from 29,205 eV (at −1.15 V) to 29203.6 at –1.55 and 29202.7 eV at –1.70 V. Their measurement values indicated that theSnO_2_NPs@rGO is stable at −1.15 V and was found to gradually reduce to lower oxidation states at higher negative polarizations. Further study with EXAFS measurements supported the above observations. EXAFS spectra at −1.55 V showed an additional peak corresponding to the Sn-Sn back scattering in the metallic Sn, and the intensity of this peak was found to increase at −1.70 V, also the spectral signatures corresponding to Sn-O bond was found to decrease. EXAFS measurements together with the recorded XANES spectra confirm the reduction of SnO_2_NPs@rGO catalyst to metallic Sn during the cathodic bias above −1.15 V. This result is crucial in the catalysis of electrochemical CO_2_ reduction as the Sn(II) oxidation state is the active species for the CO_2_ reduction to yield formate, and the metallic form of Sn is active for HER. The group has further extended and supported their study with the insights from operando-Raman spectroscopy which is far more sensitive toward the surface of the catalyst (Dutta et al., 2018).

#### Near Ambient Pressure X-Ray Photoelectron Spectroscopy

X-ray photoelectron spectroscopy (XPS) is one of the finest analytical methods to investigate the composition and electronic structures of solid materials. XPS analysis normally employs ultrahigh vacuum (UHV) conditions; this imposes the practical limitation to make use of the technique in real time analysis of catalytic surfaces during the course of reaction. Hence the most XPS characterizations have been done *ex-situ* on the immersed electrode surfaces before and after the electrochemical reaction (De Bruijn et al., 1992; He et al., 2001; Kónya et al., 2001). With modern advancements, specialized XPS systems can operate today at relatively higher pressures. The near-ambient pressure XPS (AP-XPS) reduces the challenges inherent in *ex-situ* studies and allows characterization of the catalyst under working conditions (Temperton et al., 2019; Zhong et al., 2019). In an XPS experiment, the X-ray radiation is irradiated on the sample, and the energy of ejected photoelectron is measured using an electron energy analyzer. Einstein's photoelectric equation is applied to calculate the binding energy of the ejected electron from the energy of incident X-ray radiation. As the ejected photoelectrons are of low energy, only those from the outer atomic layers are spotted without energy loss, making the XPS studies constricted to the surface of the material (Handoko et al., 2018; Zhong et al., 2019). Erenet al., investigated the Cu single crystals to explore the effect of surface planes in electrochemical CO_2_ reduction. The study particularly focused on the interaction of CO_2_ with (111) and (100) planes of Cu metal. The measured O1s spectrum showed two peaks corresponding to the dissociated surface atomic oxygen and oxygen of $C{O_{2}}^{-}$ species, and the C1s spectrum displayed the peaks corresponding to hydrocarbon and $C{O_{2}}^{-}$ entities (Eren et al., 2016). Combined investigation with scanning tunneling microscopy (STM) have shown that the surface atomic oxygen coverage is high on the Cu (100) plane along with a small coverage of $C{O_{2}}^{-}$, which confirmed that the CO_2_ dissociation is active on the Cu (100) plane, which is known to have lower planar density and four-fold adsorption sites compared to the (111) plane that is more coordinatively saturated with three-fold type adsorption sites. Yu et al. performed operando XPS studies on ceria toward CO_2_RR in a solid oxide fuel cell (Yu et al., 2014). The results revealed that during positive bias the $CO_{3}^{2-}$concentration increase, which is accompanied by Ce^3+^/Ce^4+^ redox changes, followed by the reduction of $CO_{3}^{2-}$ by Ce^3+^ to yield CO, and O^2−^ ions. The increase in the steady-state concentration of ${\text{CO}}_{3}^{2-}$ implied that the coordination of CO_2_ on the ceria surface to form a ${\text{CO}}_{3}^{2-}$ intermediate precedes as the rate limiting step. Permyakova et al., carried out XPS studies on the oxidation state of Cu_2_O during the electrochemical reduction of CO_2_. Analysis showed a complete reduction of Cu_2_O present on the surface (Permyakova et al., 2019).

#### Summary and Outlook

Electroreduction of CO_2_ is one of the most sought-after energy conversion reactions, as it can yield useful organics such as formic acid, methane, methanol and dimethyl ether at near ambient conditions. CO_2_ sequestration is one solution, but it is likely to have an energy-intensive cost. The key challenge in finding appropriate electrocatalysts for the reduction of CO_2_ is essential since the evolution of H_2_ and reduction of CO_2_ take place in parallel. The activity of an electrocatalyst toward electrochemical reduction of CO_2_ cannot be measured through comparative voltammetry alone. Consequently, the interpretation of electrochemical data by itself can be ambiguous and misleading. Therefore, electrochemical CO_2_ reduction to $C{O_{2}}^{-}$combined with optical and X-ray techniques would provide insights on the influence of the electronic and geometric effects, making it possible to deduce how intermediate products in the reduction of carbon dioxide, such as carboxylic acid and carbon monoxide, will interact with the surface of a newly proposed catalyst and thereby provide the means for predicting the catalyst's performance.

## Author Contributions

All authors listed have made a substantial, direct and intellectual contribution to the work, and approved it for publication.

### Conflict of Interest

The authors declare that the research was conducted in the absence of any commercial or financial relationships that could be construed as a potential conflict of interest.

## References

[B1] Alper E. Yuksel Orhan O. (2017). CO_2_ utilization: developments in conversion processes. Petroleum 3, 109–126. 10.1016/j.petlm.2016.11.003

[B2] Angamuthu R. Byers P. Lutz M. Spek A. L. Bouwman E. (2010). Electrocatalytic CO_2_ conversion to oxalate by a copper complex. Science 327, 313–315. 10.1126/science.1177981 20075248

[B3] Bandarenka A. S. Ventosa E. Maljusch A. Masa J. Schuhmann W. (2014). Techniques and methodologies in modern electrocatalysis: evaluation of activity, selectivity and stability of catalytic materials. Analyst 139, 1274–1291. 10.1039/c3an01647a 24418971

[B4] Bergmann A. Roldan Cuenya B. (2019). Operando insights into nanoparticle transformations during catalysis. ACS Catal. 9, 10020–43. 10.1021/acscatal.9b01831

[B5] Blanco D. E. Modestino M. A. (2019). Organic electrosynthesis for sustainable chemical manufacturing. Trends Chem. 1, 8–10. 10.1016/j.trechm.2019.01.001

[B6] Bunker G. (2010). Introduction to XAFS: A Practical Guide to X-ray Absorption Fine Structure Spectroscopy. Cambridge: Cambridge University Press 10.1017/CBO9780511809194

[B7] Cao L. Sun C. Sun N. Meng L. Chen D. (2013). Theoretical mechanism studies on the electrocatalytic reduction of CO_2_ to formate by water-stable iridium dihydride pincer complex. Dalt. Trans. 42, 5755–5763. 10.1039/c3dt32984d 23450254

[B8] Cheng T. Xiao H. Goddard W. A. (2016). Reaction mechanisms for the electrochemical reduction of CO_2_ to CO and formate on the Cu(100) surface at 298 K from quantum mechanics free energy calculations with explicit water. J. Am. Chem. Soc. 138, 13802–13805. 10.1021/jacs.6b08534 27726392

[B9] Choi Y. W. Mistry H. Roldan Cuenya B. (2017). New insights into working nanostructured electrocatalysts through operando spectroscopy and microscopy. Curr. Opin. Electrochem. 1, 1–148. 10.1016/j.coelec.2017.01.004

[B10] Christensen P. Hamnett A. (2000). *In-situ* techniques in electrochemistry - ellipsometry and FTIR. Electrochim. Acta 45, 2443–2459. 10.1016/S0013-4686(00)00332-7

[B11] Cui H. Guo Y. Guo L. Wang L. Zhou Z. Peng Z. (2018). Heteroatom-doped carbon materials and their composites as electrocatalysts for CO_2_ reduction. J. Mater. Chem. A 6, 18782–18793. 10.1039/C8TA07430E

[B12] Dahn J. R. Mao O. (1999). Application of *in situ* mössbauer effect methods for the study of electrochemical reactions in lithium-ion battery electrode materials. Phys. Rev. B Condens. Matter Mater. Phys. 59, 3494–3500. 10.1103/PhysRevB.59.3494

[B13] Dai L. Xue Y. Qu L. Choi H. J. Baek J. B. (2015). Metal-free catalysts for oxygen reduction reaction. Chem. Rev. 115, 4823–4892. 10.1021/cr5003563 25938707

[B14] De Bruijn F. A. Marin G. B. Niemantsverdriet J. W. Visscher W. H. M. Van Veen J. A. R. (1992). Characterization of graphite-supported platinum catalysts by electrochemical methods and XPS. Surf. Interface Anal. 19, 537–542. 10.1002/sia.7401901100

[B15] Deng W. Zhang L. Li L. Chen S. Hu C. Zhao Z. J.. (2019). Crucial role of surface hydroxyls on the activity and stability in electrochemical CO_2_ reduction. J. Am. Chem. Soc. 141, 2911–2915. 10.1021/jacs.8b13786 30715865

[B16] Durst J. Siebel A. Simon C. Hasché F. Herranz J. Gasteiger H. A. (2014). New insights into the electrochemical hydrogen oxidation and evolution reaction mechanism. Energy Environ. Sci. 7, 2255–2260. 10.1039/C4EE00440J

[B17] Dutta A. Kuzume A. Kaliginedi V. Rahaman M. Sinev I. Ahmadi M. (2018). Probing the chemical state of tin oxide NP catalysts during CO_2_ electroreduction: a complementary operando approach. Nano Energy 53, 828–840. 10.1016/j.nanoen.2018.09.033

[B18] Eftekhari A. (2017). Electrocatalysts for hydrogen evolution reaction ScienceDirect Electrocatalysts for hydrogen evolution reaction. Int. J. Hydrogen Energy 42, 11053–11077. 10.1016/j.ijhydene.2017.02.125

[B19] Eilert A. Roberts F. S. Friebel D. Nilsson A. (2016). Formation of copper catalysts for CO_2_ reduction with high ethylene/methane product ratio investigated with *in situ* X - ray absorption spectroscopy. J. Phys. Chem. Lett. 7, 1466–1470. 10.1021/acs.jpclett.6b00367 27045045

[B20] Eren B. Weatherup R. S. Liakakos N. Somorjai G. A. Salmeron M. (2016). Dissociative carbon dioxide adsorption and morphological changes on Cu(100) and Cu(111) at ambient pressures. J. Am. Chem. Soc. 138, 8207–8211. 10.1021/jacs.6b04039 27280375

[B21] Eslamibidgoli M. J. Huang J. Kadyk T. Malek A. Eikerling M. (2016). How theory and simulation can drive fuel cell electrocatalysis. Nano Energy 29, 334–361. 10.1016/j.nanoen.2016.06.004

[B22] Firet N. J. Blommaert M. A. Burdyny T. Venugopal A. Bohra D. Longo A. (2019). Operando EXAFS study reveals presence of oxygen in oxide-derived silver catalysts for electrochemical CO_2_ reduction. J. Mater. Chem. A 7, 2597–2607. 10.1039/C8TA10412C

[B23] Franzen D. Ellendorff B. Paulisch M. C. Hilger A. Osenberg M. Manke I. (2019). Influence of binder content in silver-based gas diffusion electrodes on pore system and electrochemical performance. J. Appl. Electrochem. 49, 705–713. 10.1007/s10800-019-01311-4

[B24] Gao D. Zhang Y. Zhou Z. Cai F. Zhao X. Huang W.. (2017a). Enhancing CO_2_ electroreduction with the metal-oxide interface. J. Am. Chem. Soc. 139, 5652–5655. 10.1021/jacs.7b00102 28391686

[B25] Gao D. Zhou H. Cai F. Wang D. Hu Y. Jiang B. (2017b). Switchable CO_2_ electroreduction via engineering active phases of Pd nanoparticles. Nano Res. 10, 2181–2191. 10.1007/s12274-017-1514-6

[B26] Genovese C. Schuster M. E. Gibson E. K. Gianolio D. Posligua V. Grau-Crespo R.. (2018). Operando spectroscopy study of the carbon dioxide electro-reduction by iron species on nitrogen-doped carbon. Nat. Commun. 9, 1–12. 10.1038/s41467-018-03138-7 29507285 PMC5838105

[B27] Grosjean R. Delacroix S. Gouget G. Beaunier P. Ersen O. Ihiawakrim D. (2017). High pressures pathway toward boron-based nanostructured solids. Dalt. Trans. 47, 7634–7639. 10.1039/C8DT00932E 29796509

[B28] Grosse P. Gao D. Scholten F. Sinev I. Mistry H. Roldan Cuenya B. (2018). Dynamic changes in the structure, chemical state and catalytic selectivity of Cu nanocubes during CO_2_ electroreduction: size and support effects. Angew. Chemie Int. Ed. 57, 6192–6197. 10.1002/anie.201802083 29578622

[B29] Handoko A. D. Wei F. Jenndy Yeo B. S. Seh Z. W. (2018). Understanding heterogeneous electrocatalytic carbon dioxide reduction through operando techniques. Nat. Catal. 1, 922–934. 10.1038/s41929-018-0182-6

[B30] He F. Liu C. J. Eliasson B. Xue B. (2001). XPS characterization of zeolite catalyst in plasma catalytic methane conversion. Surf. Interface Anal. 32, 198–201. 10.1002/sia.1036

[B31] Hori Y. Takahashi R. Yoshinami Y. Murata A. (1997). Electrochemical reduction of CO at a copper electrode. J. Phys. Chem. B 101, 7075–7081. 10.1021/jp970284i

[B32] Hori Y. Wakebe H. Tsukamoto T. Koga O. (1994). Electrocatalytic Process of CO selectivity in electrochemical reduction of CO_2_ at metal electrodes in aqueous media. Electrochim. Acta 39, 1833–1839. 10.1016/0013-4686(94)85172-7

[B33] Jiang H. He Q. Zhang Y. Song L. (2018). Structural self-reconstruction of catalysts in electrocatalysis. Acc. Chem. Res. 51, 2968–2977. 10.1021/acs.accounts.8b00449 30375841

[B34] Jiang K. Wang H. Cai W. Bin Wang H. (2017). Li electrochemical tuning of metal oxide for highly selective CO_2_ reduction. ACS Nano 11, 6451–6458. 10.1021/acsnano.7b03029 28558186

[B35] Kas R. Ayemoba O. Firet N. J. Middelkoop J. Smith W. A. Cuesta A. (2019). *In-Situ* infrared spectroscopy applied to the study of the electrocatalytic reduction of CO_2_: theory, practice and challenges. ChemPhysChem. 20, 2904–2925. 10.1002/cphc.201900533 31441195

[B36] König M. Vaes J. Klemm E. Pant D. (2019). Solvents and supporting electrolytes in the electrocatalytic reduction of CO_2_. Science 19, 135–160. 10.1016/j.isci.2019.07.014 31369986 PMC6669325

[B37] Kónya Z. Kiss J. Oszkó A. Siska A. Kiricsi I. (2001). XPS characterisation of catalysts during production of multiwalled carbon nanotubes. Phys. Chem. Chem. Phys. 3, 155–158. 10.1039/b007279f

[B38] Kotrel S. Bräuninger S. (2008). Industrial electrocatalysis, in Handbook of Heterogeneous Catalysis (Wiley), 1936–1958. 10.1002/9783527610044.hetcat0103

[B39] Li J. T. Zhou Z. Y. Broadwell I. Sun S. G. (2012). *In-situ* infrared spectroscopic studies of electrochemical energy conversion and storage. Acc. Chem. Res. 45, 485–494. 10.1021/ar200215t 22264174

[B40] Li W. Seredych M. Rodríguez-Castellón E. Bandosz T. J. (2016). Metal-free nanoporous carbon as a catalyst for electrochemical reduction of CO_2_ to CO and CH_4_. ChemSusChem 9, 606–616. 10.1002/cssc.201501575 26835880

[B41] Narayanan R. Basuri P. Jana S. K. Mahendranath A. Bose S. Pradeep T. (2019). *In situ* monitoring of electrochemical reactions through CNT-assisted paper cell mass spectrometry. Analyst 144, 5404–5412. 10.1039/C9AN00791A 31363725

[B42] Nelson R. C. Miller J. T. (2012). An introduction to X-ray absorption spectroscopy and its *in situ* application to organometallic compounds and homogeneous catalysts. Catal. Sci. Technol. 2, 461–470. 10.1039/C2CY00343K

[B43] Neubrech F. Huck C. Weber K. Pucci A. Giessen H. (2017). Surface-enhanced infrared spectroscopy using resonant nanoantennas. Chem. Rev. 117, 5110–5145. 10.1021/acs.chemrev.6b00743 28358482

[B44] Oda I. Ogasawara H. Ito M. (1996). Carbon monoxide adsorption on copper and silver electrodes during carbon dioxide electroreduction studied by infrared reflection absorption spectroscopy and surface-enhanced raman spectroscopy. Langmuir 12, 1094–1097. 10.1021/la950167j

[B45] Oh Y. Vrubel H. Guidoux S. Hu X. (2014). Electrochemical reduction of CO_2_ in organic solvents catalyzed by MoO_2_. Chem. Commun. 50, 3878–3881. 10.1039/c3cc49262a 24589502

[B46] Ooka H. Figueiredo M. C. Koper M. T. M. (2017). Competition between hydrogen evolution and carbon dioxide reduction on copper electrodes in mildly acidic media. Langmuir 33, 9307–9313. 10.1021/acs.langmuir.7b00696 28453940 PMC5607460

[B47] Papasizza M. Cuesta A. (2018). *In situ* monitoring using ATR-SEIRAS of the electrocatalytic reduction of CO_2_ on Au in an ionic liquid/water mixture. ACS Catal. 8, 6345–6352. 10.1021/acscatal.8b00977

[B48] Pérez-Rodríguez S. García G. Lázaro M. J. Pastor E. (2018). Chapter 9: probing CO_2_ reduction intermediates employing *in situ* spectroscopy and spectrometry, in Electrochemical Reduction of Carbon Dioxide: Overcoming the Limitations of Photosynthesis, eds Marken F. Fermin D. (Santa Cruz de Tenerife: RSC,Energy and Environment Series), 212–243. 10.1039/9781782623809-00212

[B49] Permyakova A. A. Herranz J. El Kazzi M. Diercks J. S. Povia M. Mangani L. R. (2019). On the oxidation state of Cu_2_O upon electrochemical CO_2_ reduction: an XPS study. ChemPhysChem 20, 3120–3127. 10.1002/cphc.201900468 31310028

[B50] Qiao J. Liu Y. Zhang J. (eds.). (2016). Electrochemical Reduction of Carbon Dioxide. Boca Raton, FL: CRC Press 10.1201/b20177

[B51] Raciti D. Wang C. (2018). Recent advances in CO_2_ reduction electrocatalysis on copper. ACS Energy Lett. 3, 1545–1556. 10.1021/acsenergylett.8b00553

[B52] Rasul S. Pugnant A. Xiang H. Fontmorin J. M. Yu E. H. (2019). Low cost and efficient alloy electrocatalysts for CO_2_ reduction to formate. J. CO_2_ Util. 32, 1–10. 10.1016/j.jcou.2019.03.016

[B53] Rehr J. J. Kas J. J. Vila F. D. Prange M. P. Jorissen K. (2010). Parameter-free calculations of X-ray spectra with FEFF9. Phys. Chem. Chem. Phys. 12, 5503–5513. 10.1039/b926434e 20445945

[B54] Rodes A. Pastor E. Iwasita T. (1994a). Structural effects on CO_2_ reduction at Pt single-crystal electrodes: part 1. The Pt(110) surface. J. Electroanal. Chem. 369, 183–191. 10.1016/0022-0728(94)87097-7

[B55] Rodes A. Pastor E. Iwasita T. (1994b). Structural effects on CO_2_ reduction at Pt single-crystal electrodes: part 2. Pt(111) and vicinal surfaces in the [011] zone. J. Electroanal. Chem. 373, 167–175. 10.1016/0022-0728(94)03306-4

[B56] Rodes A. Pastor E. Iwasita T. (1994c). Structural effects on CO_2_ reduction at Pt single-crystal electrodes: part 3. Pt(100) and related surfaces. J. Electroanal. Chem. 377, 215–225. 10.1016/0022-0728(94)03424-9

[B57] Sapountzi F. M. Gracia J. M. Weststrate C. J. Kee J. Fredriksson H. O. A. Niemantsverdriet J. W. (2017). Electrocatalysts for the generation of hydrogen, oxygen and synthesis gas. Prog. Energy Combust. Sci. 58, 1–35. 10.1016/j.pecs.2016.09.001

[B58] Sen S. Liu D. Palmore G. T. R. (2014). Electrochemical reduction of CO2 at copper nanofoams. ACS Catalysis 4, 3091–3095. 10.1021/cs500522g

[B59] Shao M. Chang Q. Dodelet J. P. Chenitz R. (2016). Recent advances in electrocatalysts for oxygen reduction reaction. Chem. Rev. 116, 3594–3657. 10.1021/acs.chemrev.5b00462 26886420

[B60] Singh J. Lamberti C. Van Bokhoven J. A. (2010). Advanced X-ray absorption and emission spectroscopy: *in situ* catalytic studies. Chem. Soc. Rev. 39, 4754–4766. 10.1039/c0cs00054j 20981379

[B61] Skúlason E. Tripkovic V. Björketun M. E. Gudmundsdóttir S. Karlberg G. Rossmeisl J. (2010). Modeling the electrochemical hydrogen oxidation and evolution reactions on the basis of density functional theory calculations. J. Phys. Chem. C 114, 18182–18197. 10.1021/jp1048887

[B62] Smith B. C. (2011). Fundamentals of Fourier Transform Infrared Spectroscopy, 2 Edn. Boca Raton, FL: CRC Press, Taylor & Francis Group 10.1201/b10777

[B63] Sullivan B. P. Krist K. Guard H. E. (eds.). (1993). Electrochemical and Electrocatalytic Reactions of Carbon Dioxide. Elsevier Science Publishers B.V 10.1016/b978-0-444-88316-2.50001-2

[B64] Sun S. Christensen P. A. Wieckowski A. (2007). In-situ Spectroscopic Studies of Adsorption at the Electrode and Electrocatalysis, 1st Edn. Elsevier.

[B65] Sun Z. Ma T. Tao H. Fan Q. Han B. (2017). Fundamentals and challenges of electrochemical CO_2_ reduction using two-dimensional materials. Chem 3, 560–587. 10.1016/j.chempr.2017.09.009

[B66] Surca A. K. Rodošek M. Kreta A. Mihelčič M. Gaberšček, M. (2017). *In situ* and *ex situ* electrochemical measurements: spectroelectrochemistry and atomic force microscopy, in Hybrid Organic-Inorganic Interfaces: Towards Advanced Functional Materials, eds Delville M.-H. Taubert A. (Wiley). 10.1002/9783527807130.ch19

[B67] Tang S. Zhou X. Zhang S. Li X. Yang T. Hu W.. (2019). Metal-free boron nitride nanoribbon catalysts for electrochemical CO_2_ reduction: combining high activity and selectivity. ACS Appl. Mater. Interfaces 11, 906–915. 10.1021/acsami.8b18505 30525373

[B68] Temperton R. H. Gibson A. O'Shea J. N. (2019). In situ XPS analysis of the atomic layer deposition of aluminium oxide on titanium dioxide. Phys. Chem. Chem. Phys. 21, 1393–1398. 10.1039/C8CP06912C 30601499

[B69] Velasco-Vélez J. J. Jones T. Gao D. Carbonio E. Arrigo R. Hsu C. J. (2019). The role of the copper oxidation state in the electrocatalytic reduction of CO_2_ into valuable hydrocarbons. ACS Sustain. Chem. Eng. 7, 1485–1492. 10.1021/acssuschemeng.8b05106

[B70] Wang M. Árnadóttir L. Xu Z. J. Feng Z. (2019). *In situ* X-ray absorption spectroscopy studies of nanoscale electrocatalysts. Nano-Micro Lett. 11:47 10.1007/s40820-019-0277-x PMC7770664 34138000

[B71] Weng Z. Wu Y. Wang M. Jiang J. Yang K. Huo S.. (2018). Active sites of copper-complex catalytic materials for electrochemical carbon dioxide reduction. Nat. Commun. 9, 1–9. 10.1038/s41467-018-02819-7 29379087 PMC5788987

[B72] Wu Y. Jiang J. Weng Z. Wang M. Broere D. L. J. Zhong Y.. (2017). Electroreduction of CO_2_ catalyzed by a heterogenized Zn-porphyrin complex with a redox-innocent metal center. ACS Cent. Sci. 3, 847–852. 10.1021/acscentsci.7b00160 28852698 PMC5571454

[B73] Wuttig A. Liu C. Peng Q. Yaguchi M. Hendon C. H. Motobayashi K.. (2016). Tracking a common surface-bound intermediate during CO_2_-to-fuels catalysis. ACS Cent. Sci. 2, 522–528. 10.1021/acscentsci.6b00155 27610413 PMC4999975

[B74] Xu H. Ci S. Ding Y. Wang G. Wen Z. (2019). Recent advances in precious metal-free bifunctional catalysts for electrochemical conversion systems. J. Mater. Chem. A 7, 8006–8029. 10.1039/C9TA00833K

[B75] Yajima T. Uchida H. Watanabe M. (2004). *In-situ* ATR-FTIR spectroscopic study of electro-oxidation of methanol and adsorbed CO at Pt-Ru alloy. J. Phys. Chem. B. 108, 2654–2659. 10.1021/jp037215q

[B76] Ye J. Y. Jiang Y. X. Sheng T. Sun S. G. (2016). *In-situ* FTIR spectroscopic studies of electrocatalytic reactions and processes. Nano Energy 29, 414–427. 10.1016/j.nanoen.2016.06.023

[B77] Yin Z. Palmore G. T. R. Sun S. (2019). Electrochemical reduction of CO_2_ catalyzed by metal nanocatalysts. Trends Chem. 1, 1–12. 10.1016/j.trechm.2019.05.004

[B78] Yu F. Wei P. Yang Y. Chen Y. Guo L. Peng Z. (2019). Material design at nano and atomic scale for electrocatalytic CO2 reduction. Nano Mater. Sci. 1, 60–69. 10.1016/j.nanoms.2019.03.006

[B79] Yu Y. Mao B. Geller A. Chang R. Gaskell K. Liu Z.. (2014). CO_2_ activation and carbonate intermediates: an *operando* AP-XPS study of CO_2_ electrolysis reactions on solid oxide electrochemical cells. Phys. Chem. Chem. Phys. 16, 11633–11639. 10.1039/C4CP01054J 24806971

[B80] Zeng M. Li Y. (2015). Recent advances in heterogeneous electrocatalysts for the hydrogen evolution reaction. J. Mater. Chem. A 3, 14942–14962. 10.1039/C5TA02974K

[B81] Zhang G. Yuan J. Liu Y. Lu W. Fu N. Li W. (2018). Boosting the oxygen evolution reaction in non-precious catalysts by structural and electronic engineering. J. Mater. Chem. A 6, 10253–10263. 10.1039/C8TA02542H

[B82] Zhong L. Chen D. Zafeiratos S. (2019). A mini review of *in situ* near-ambient pressure XPS studies on non-noble, late transition metal catalysts. Catal. Sci. Technol. 9, 3851–3867. 10.1039/C9CY00632J

[B83] Zhu K. Zhu X. Yang W. (2019). Application of *in situ* techniques for the characterization of NiFe-based oxygen evolution reaction (OER) electrocatalysts. Angew. Chemie Int. Ed. 58, 1252–1265. 10.1002/anie.201802923 29665168

[B84] Zhu S. Jiang B. Cai W-B. Shao M. (2017). Direct observation on reaction intermediates and the role of bicarbonate anions in CO_2_ electrochemical reduction reaction on Cu surfaces. J. Am. Chem. Soc. 139, 15664–15667. 10.1021/jacs.7b10462 29058890

[B85] Zhu S. Li T. Cai W. B. Shao M. (2019). CO_2_ electrochemical reduction as probed through infrared spectroscopy. ACS Energy Lett. 4, 682–689. 10.1021/acsenergylett.8b02525

